# A Ketogenic Diet May Offer Neuroprotection in Glaucoma and Mitochondrial Diseases of the Optic Nerve

**Published:** 2012

**Authors:** Tomasz Zarnowski, Maria Tulidowicz-Bielak, Ewa Kosior-Jarecka, Iwona Zarnowska, Waldemar A. Turski, Maciej Gasior

**Affiliations:** 1 Chair of Ophthalmology, Medical University, Lublin, Poland; 2 SANITAS Private Medical Center, Lublin, Poland; 3 Department of Experimental and Clinical Pharmacology, Medical University, Lublin, Poland; 4 Department of Pharmacology and Physiology, Drexel University College of Medicine, Philadelpia, USA

**Keywords:** Glaucoma, Ketogenic diet, Optic nerve diseases, Neuroprotection, Mitochondrial disease

## Abstract

Glaucoma is a chronic optic nerve disease in which the primary damage occurs to the retinal ganglion cell axons. Therapies that prevent the death of retinal ganglion cells should be theoretically beneficial. Despite promising preclinical studies, however, almost all clinical studies with pharmacological approaches for neuroprotection in neurologic and eye diseases, including glaucoma, have so far failed to show efficacy. As the evidence supporting the neuroprotective efficacy of a ketogenic diet (KD) in a number of neurodegenerative diseases continues to grow, it is conceivable that this metabolic approach might be useful in chronic glaucoma. Putative cellular mechanisms underlying the neuroprotective activity of the KD have been identified in neurological studies, including effects on energy metabolism, the GABA system, glutamate-mediated toxicity, antioxidant mechanisms, programmed cell death, anti-inflammatory mechanisms, and the production of kynurenic acid. Of note, the same mechanisms are thought to be involved in glaucoma. Given these mechanistic similarities, testing the KD for its efficacy in neurodegenerative diseases of the eye is proposed.

## INTRODUCTION

Glaucoma is the most common optic neuropathy and the second most common cause of blindness worldwide [1]. Primary open-angle glaucoma (POAG) is the most frequent type of glaucoma, with risk factors that include elevated intraocular pressure (IOP), family history, old age, or black racial ancestry [2]. Although the primary pathogenesis of POAG remains unknown, chronic retinal ganglion cell apoptosis with consequent progressive damage to axons at the optic nerve head has been implicated [3]. At the cellular level and consistently with neurodegenerative processes of other CNS disorders [4], glutamate-mediated excitotoxicity, intracellular calcium overload, mitochondrial dysfunction, and the generation of reactive oxygen species are likely to be involved in the POAG-induced optic nerve neuropathy [3].

 There is growing evidence that inborn mitochondrial dysfunctions may play an important role in a large proportion of patients affected by POAG. Results of a recent study reveal a spectrum of mitochondrial abnormalities in patients with POAG, implicating oxidative stress and suggesting that mitochondrial dysfunction may be a risk factor for POAG [5]. The development of glaucomatous damage in that subpopulation of patients often could not be explained by risk factors typically associated with POAG, such as family history, systemic risk factors, or high IOP. Thus, studies focusing on the association between mitochondrial function and glaucoma open a novel research approach into the pathologies and potential treatments of POAG-related optic neuropathies [6]. 

The list of optic neuropathies already proven to be associated with mitochondrial abnormalities can be seen in patients with Leber’s hereditary optic neuropathy (LHON), or in certain patients with optic neuritis, multiple sclerosis, Wolfram’s syndrome, dominant optic atrophy, or non-arteritic ischaemic optic neuropathy [7,8]. In contrast to chronic POAG, retinal ganglion cell apoptosis and axonal injury at the optic nerve head in LHON is subacute [7]. Interestingly, in this mtDNA-mediated disorder, normal tension glaucoma of an unknown pathomechanism appears to be present more often [9]. However, the exact mechanisms by which mitochondrial abnormalities put the optic nerve at risk remain uncertain. It is possible that a high concentration of mitochondria at the optic nerve head implies the dependency of neuronal survival on mitochondrial function [7,8].

Despite a tremendous effort over the last two decades to establish effective neuroprotective treatments in glaucoma and other neuropathies, there are no novel treatments beyond those that target lowering the IOP. This approach, unfortunately, often turns out to be unsuccessful; thus suggesting the involvement of other factors/mechanisms in the pathogenesis and/or progression of neurodegenerative diseases of the eye [10]. 

For nearly 90 years, the ketogenic diet (KD) has been used successfully to treat patients with intractable epilepsy. It was introduced in clinical use to mimic biochemical changes that occur during starvation [11]. The KD is high in fats and low in carbohydrates and proteins [12]. During prolonged exposure to the KD, energy is mainly derived from the oxidation of fatty acids in mitochondria as opposed to glucose being the main energy source when exposed to a normal diet. When exposed to the KD, fatty acids are oxidised at a high rate, which results in an overproduction of acetyl-CoA. The overproduction of acetyl-CoA then leads to the synthesis of ketone bodies, such as β-hydroxybutyrate, acetoacetate, and acetone, primarily in the liver [12]. These ketone bodies serve as energy substrates and there is evidence that they can enhance neuronal survival under some pathological conditions, including hypoxia, anoxia, or ischaemia [13]. It has recently been shown that acetoacetate and β-hydroxybutyrate produced a significant dose-dependent neuroprotective effect on retinal ganglion cells in a rat model of NMDA-induced neuronal damage [14]. The KDs have been shown to exert neuroprotective effects in brain trauma [15], Alzheimer's disease [16], Parkinson’s disease [17], and amyotrophic lateral sclerosis [18]. Also, the anticonvulsant effects of KDs have been well documented [19, 20], which is consistent with its therapeutic use in the treatment of refractory epilepsy [21,22].

## HYPOTHESIS

It is proposed that the KD may have a therapeutic benefit in diseases of the eye associated with neurodegeneration.

## DISCUSSION

When chronically exposed to the KD, the metabolic efficiency of the Kreb’s cycle is reduced and the overproduced acetyl-CoA is shunted to the production of ketone bodies that are then utilised as an energy source in extra-hepatic tissues, including the brain. Glucose is normally the sole energy source for the human brain, as fatty acids cannot be used because they do not cross the blood-brain barrier. Ketone bodies do enter the brain, in proportion to the degree of ketosis. Under normal conditions, when carbohydrates are abundant, the utilisation of ketones by the brain is minimal. When exposed to the KD, however, ketone bodies become the major energy source for the brain. The ketone bodies are converted to acetyl-CoA by D--hydroxybutyrate dehydrogenase, acetoacetate-succinyl-CoA transferase and acetoacetyl-CoA-thiolase, and then enter the Kreb’s cycle within the mitochondria of the brain to produce ATP [12].

It has been known since the time of Hippocrates that fasting is an effective treatment for seizures, and approaches utilising the KD were designed to mimic the fasting state [23]. Despite intensive research over recent years, the mechanism by which the KD affects seizures remains unknown. The diet is associated with a wide range of neurochemical changes, some of which may contribute to its therapeutic actions and others that are epiphenomena. The current poor understanding of the mechanisms involved in the therapeutic effects of the KD is not that different from that for many approved antiepileptic drugs (AEDs); levetiracetam can serve as a most recent example. This drug was approved many years before its mechanisms of action were discovered [24].

Although far from definitely proven, there is emerging evidence that the KD may also have disease-modifying actions in epilepsy and other diseases associated with neuronal death [25]. New findings suggest that the KD can be more beneficial for the treatment of seizures associated with metabolic stress or underlying metabolic abnormalities [26].

Much of the neurological dysfunction that occurs in stroke, cerebral ischaemia, glaucoma, or acute traumatic brain injury patients is due to secondary injury processes involving glutamate-mediated excitotoxicity, intracellular calcium overload, mitochondrial dysfunction, or the generation of reactive oxygen species (ROS) [27]. Consequently, the underlying pathophysiological mechanisms may have features in common with those in classical neurodegenerative disorders (Alzheimer’s and Parkinson’s diseases). 

Mitochondria play important cellular functions that include the production of cellular ATP, the control of apoptosis, the maintenance of calcium homeostasis, and the production and elimination of reactive oxygen species. Chronic exposure to the KD stimulates mitochondrial biogenesis, ATP production, and phosphocreatine concentrations in the brain, suggesting metabolic efficiency [28]. Furthermore, genes encoding bioenergetic enzymes are up-regulated by the KD [28, 29]. Recently, it was shown that the KD modulates oxidative stress and also increases mitochondrial glutathione levels [30,31]. All of these findings might be relevant for alleviating a chronic neurodegenerative disease, like glaucoma, that clearly exhibits features of mitochondrial dysfunction or insufficiency, as well as diseases associated with mtDNA depletion, like Leber’s hereditary optic neuropathy. 

Recently, Prins and co-workers reported that the KD can confer up to a 60% reduction in cortical contusion volume at 7 days after controlled cortical injury in rats [32]. Of note, the beneficial effects of the KD that was administered after the injury only occurred at some postnatal ages, despite the similar availability of ketone bodies at all of the studied ages. This led the authors to conclude that differences in the ability of the brain to utilise ketones at different developmental stages may affect the efficacy of ketone bodies-induced protection [33,34]. An additional study found that rats receiving the KD were also resistant to the loss of cortical neurons during insulin-induced hypoglycaemia [35]. Thus, there is evidence that the KD has neuroprotective activity in traumatic-, ischaemic-, and metabolic-related brain injuries. 

Although the mechanism by which the KD confers protection in these diverse injury models is not well understood, ketone bodies could play a direct role. For example, β-hydroxybutyrate could presumably serve as an alternative energy source to mitigate injury-induced ATP depletion. In fact, the exogenous administration of β-hydroxybutyrate has been reported to reduce brain damage and improve neuronal function in models of brain hypoxia, anoxia, and ischaemia [13,36,37,38]. Likewise, the other ketone bodies, acetoacetate and acetone, can also serve as alternative energy sources and exhibit similar neuroprotective effects [39,40,41]. Interestingly, in rats maintained on the KD, neuronal uptake of β-hydroxybutyrate was increased after cortical impact injury in comparison to animals receiving a standard diet [42], suggesting the facilitated brain penetrations of ketone bodies when chronically exposed to the KD. Moreover, it has been shown that two ketone bodies, acetoacetate and β-hydroxybutyrate, can increase the formation of brain kynurenic acid that is considered an endogenous antagonist of NMDA receptors and a putative neuroprotective agent [43,44].

The clinical application of the classical KD has been hampered by its poor tolerability and potentially serious side-effects. As the underlying mechanisms involved in the KD-induced clinical benefit become better understood, it could be possible to develop alternative therapeutic approaches with similar or improved therapeutic effects and reduced liabilities associated with chronic exposure to this high-fat diet. Alternative options of less restricted diets with presumed improved side-effect profiles and/or compliance are being investigated (e.g. the Atkins diet, low-glycaemic-index treatment or diets based on medium-chain fatty acids) [45].

Almost all of the clinical studies on neuroprotection in ophthalmologic diseases including glaucoma have so far failed to show efficacy, despite encouraging preclinical studies [46,47]. Supported by both pre-clinical and clinical evidence demonstrating the neuroprotective effects of the KD, it is reasonable to consider testing the KD in diseases of the eye associated with neurodegeneration. A properly designed multicentre study with validated endpoints would yield the most useful information in a relatively short time. 

## CONCLUSION

There is a paucity of data in the literature on the neuroprotective efficacy of the KD and ketone bodies in the neurodegeneration of retina and optic nerves. Putative cellular mechanisms underlying the neuroprotective activity of the KD have been identified in neurological studies focusing, for example, on the effects of the KD on energy metabolism, the GABA system, glutamate-mediated toxicity, antioxidant mechanisms, programmed cell death, anti-inflammatory processes, or the enhancement of kynurenic acid production. Importantly, many of these mechanisms have been implicated in glaucoma as well.

When these observations on mechanistic similarities are taken together, it raises the possibility that the KD may be neuroprotective in certain diseases of the eye such as glaucoma, mitochondrial diseases of the optic nerve, or retinal ischaemic diseases (diabetic retinopathy and retinopathy of prematurity). Proving the efficacy of the KD in these conditions would require a proof-of-concept study to be followed, if successful, by studies in a larger number of patients.

## DISCLOSURE

The authors report no conflicts of interest in this work.

## References

[1] Quigley HA (1996). Number of people with glaucoma worldwide. Br J Ophthalmol.

[2] Gherghel D, Hosking SL, Orgül S (2004). Autonomic nervous system, circadian rhythms, and primary open-angle glaucoma. Surv Ophthalmol.

[3] Nickells RW (1999). Apoptosis of retinal ganglion cells in glaucoma: an update of the molecular pathways involved in cell death. Surv Ophthalmol.

[4] Repici M, Mariani J, Borsello T (2007). Neuronal death and neuroprotection: a review. Methods Mol Biol.

[5] Abu-Amero KK, Morales J, Bosley TM (2006). Mitochondrial abnormalities in patients with primary open-angle glaucoma. Invest Ophthalmol Vis Sci.

[6] Buono LM, Foroozan R, Sergott RC, Savino PJ (2002). Is normal tension glaucoma actually an unrecognized hereditary optic neuropathy? New evidence from genetic analysis. Curr Opin Ophthalmol.

[7] Howell N (2003). LHON and other optic nerve atrophies: the mitochondrial connection. Dev Ophthalmol..

[8] Bosley TM, Abu-Amero KK, Ozand PT (2004). Mitochondrial DNA nucleotide changes in non-arteritic ischemic optic neuropathy. Neurology.

[9] Opial D, Boehnke M, Tadesse S, Lietz-Partzsch A, Flammer J, Munier F, Mermoud A, Hirano M, Flückiger F, Mojon DS (2001). Leber's hereditary optic neuropathy mitochondrial DNA mutations in normal-tension glaucoma. Graefes Arch Clin Exp Ophthalmol.

[10] Zarnowski T, Kosior-Jarecka E (2012). Progression of normal tension glaucoma in Kearns-Sayre syndrome over 10 years. Clin Experiment Ophthalmol.

[11] Wilder RM (1921). The effect of ketonemia on the course of epilepsy. Mayo Clin Proc.

[12] Freeman J, Veggiotti P, Lanzi G, Tagliabue A, Perucca E, Institute of Neurology IRCCS C (2006). Mondino Foundation. The ketogenic diet: from molecular mechanisms to clinical effects. Epilepsy Res.

[13] Suzuki M, Suzuki M, Sato K, Dohi S, Sato T, Matsuura A, Hiraide A (2001). Effect of beta-hydroxybutyrate, a cerebral function improving agent, on cerebral hypoxia, anoxia and ischemia in mice and rats. Jpn J Pharmacol.

[14] Thaler S, Choragiewicz TJ, Rejdak R, Fiedorowicz M, Turski WA, Tulidowicz-Bielak M, Zrenner E, Schuettauf F, Zarnowski T (2010). Neuroprotection by acetoacetate and β-hydroxybutyrate against NMDA-induced RGC damage in rat--possible involvement of kynurenic acid. Graefes Arch Clin Exp Ophthalmol.

[15] Prins ML, Fujima LS, Hovda DA (2005). Age-dependent reduction of cortical contusion volume by ketones after traumatic brain injury. J Neurosci Res.

[16] Van der Auwera I, Wera S, Van Leuven F, Henderson ST (2005). A ketogenic diet reduces amyloid beta 40 and 42 in a mouse model of Alzheimer's disease. Nutr Metab (Lond).

[17] Vanitallie TB, Nonas C, Di Rocco A, Boyar K, Hyams K, Heymsfield SB (2005). Treatment of Parkinson disease with diet-induced hyperketonemia: a feasibility study. Neurology.

[18] Zhao Z, Lange DJ, Voustianiouk A, MacGrogan D, Ho L, Suh J, Humala N, Thiyagarajan M, Wang J, Pasinetti GM (2006). A ketogenic diet as a potential novel therapeutic intervention in amyotrophic lateral sclerosis. BMC Neurosci.

[19] Bough KJ, Eagles DA (1999). A ketogenic diet increases the resistance to pentylenetetrazole-induced seizures in the rat. Epilepsia.

[20] Appleton DB, DeVivo DC (1974). An animal model for the ketogenic diet. Epilepsia.

[21] Freeman JM, Vining EP, Pillas DJ, Pyzik PL, Casey JC, Kelly LM (1998). The efficacy of the ketogenic diet-1998: a prospective evaluation of intervention in 150 children. Pediatrics.

[22] Neal EG, Chaffe H, Schwartz RH, Lawson MS, Edwards N, Fitzsimmons G, Whitney A, Cross JH (2008). The ketogenic diet for the treatment of childhood epilepsy: a randomised controlled trial. Lancet Neurol.

[23] Bailey EE, Pfeifer HH, Thiele EA (2005). The use of diet in the treatment of epilepsy. Epilepsy Behav.

[24] Lynch BA, Lambeng N, Nocka K, Kensel-Hammes P, Bajjalieh SM, Matagne A, Fuks B (2004). The synaptic vesicle protein SV2A is the binding site for the antiepileptic drug levetiracetam. Proc Natl Acad Sci U S A.

[25] Gasior M, Rogawski MA, Hartman AL (2006). Neuroprotective and disease-modifying effects of the ketogenic diet. Behav Pharmacol.

[26] Samoilova M, Weisspapir M, Abdelmalik P, Velumian AA, Carlen PL (2010). Chronic in vitro ketosis is neuroprotective but not anti-convulsant. J Neurochem.

[27] McIntosh TK, Saatman KE, Raghupathi R, Graham DI, Smith DH, Lee VM, Trojanowski JQ (1998). The Dorothy Russell Memorial Lecture. The molecular and cellular sequelae of experimental traumatic brain injury: pathogenetic mechanisms. Neuropathol Appl Neurobiol.

[28] Bough KJ, Wetherington J, Hassel B, Pare JF, Gawryluk JW, Greene JG, Shaw R, Smith Y, Geiger JD, Dingledine RJ (2006). Mitochondrial biogenesis in the anticonvulsant mechanism of the ketogenic diet. Ann Neurol.

[29] Noh HS, Kang SS, Kim DW, Kim YH, Park CH, Han JY, Cho GJ, Choi WS (2005). Ketogenic diet increases calbindin-D28k in the hippocampi of male ICR mice with kainic acid seizures. Epilepsy Res.

[30] Milder J, Patel M (2012). Modulation of oxidative stress and mitochondrial function by the ketogenic diet. Epilepsy Res.

[31] Jarrett SG, Milder JB, Liang LP, Patel M (2008). The ketogenic diet increases mitochondrial glutathione levels. J Neurochem.

[32] Prins ML, Fujima LS, Hovda DA (2005). Age-dependent reduction of cortical contusion volume by ketones after traumatic brain injury. J Neurosci Res.

[33] Rafiki A, Boulland JL, Halestrap AP, Ottersen OP, Bergersen L (2003). Highly differential expression of the monocarboxylate transporters MCT2 and MCT4 in the developing rat brain. Neuroscience..

[34] Vannucci SJ, Simpson IA (2003). Developmental switch in brain nutrient transporter expression in the rat. Am J Physiol Endocrinol Metab.

[35] Yamada KA, Rensing N, Thio LL (2005). Ketogenic diet reduces hypoglycemia-induced neuronal death in young rats. Neurosci Lett.

[36] Cherian L, Peek K, Robertson CS, Goodman JC, Grossman RG (1994). Calorie sources and recovery from central nervous system ischemia. Crit Care Med.

[37] Dardzinski BJ, Smith SL, Towfighi J, Williams GD, Vannucci RC, Smith MB (2000). Increased plasma beta-hydroxybutyrate, preserved cerebral energy metabolism, and amelioration of brain damage during neonatal hypoxia ischemia with dexamethasone pretreatment. Pediatr Res.

[38] Suzuki M, Suzuki M, Kitamura Y, Mori S, Sato K, Dohi S, Sato T, Matsuura A, Hiraide A (2002). Beta-hydroxybutyrate, a cerebral function improving agent, protects rat brain against ischemic damage caused by permanent and transient focal cerebral ischemia. Jpn J Pharmacol.

[39] Massieu L, Del Río P, Montiel T (2001). Neurotoxicity of glutamate uptake inhibition in vivo: correlation with succinate dehydrogenase activity and prevention by energy substrates. Neuroscience..

[40] Massieu L, Haces ML, Montiel T, Hernández-Fonseca K (2003). Acetoacetate protects hippocampal neurons against glutamate-mediated neuronal damage during glycolysis inhibition. Neuroscience.

[41] Noh HS, Hah YS, Nilufar R, Han J, Bong JH, Kang SS, Cho GJ, Choi WS (2006). Acetoacetate protects neuronal cells from oxidative glutamate toxicity. J Neurosci Res.

[42] Prins ML, Lee SM, Fujima LS, Hovda DA (2004). Increased cerebral uptake and oxidation of exogenous betaHB improves ATP following traumatic brain injury in adult rats. J Neurochem.

[43] Hodgkins PS, Schwarcz R (1998). Interference with cellular energy metabolism reduces kynurenic acid formation in rat brain slices: reversal by lactate and pyruvate. Eur J Neurosci.

[44] Hodgkins PS, Schwarcz R (1998). Metabolic control of kynurenic acid formation in the rat brain. Dev Neurosci.

[45] Kessler SK, Neal EG, Camfield CS, Kossoff EH (2011). Dietary therapies for epilepsy: future research. Epilepsy Behav.

[46] Danesh-Meyer HV, Levin LA (2009). Neuroprotection: extrapolating from neurologic diseases to the eye. Am J Ophthalmol.

[47] Sena DF, Ramchand K, Lindsley K (2010). Neuroprotection for treatment of glaucoma in adults. Cochrane Database Syst Rev.

